# Prognosis of Chancre with Reference to the Probabilities of Secondary Symptoms

**Published:** 1845-07

**Authors:** 

**Affiliations:** Paris


*Prognosis of Chancre with reference to the probabilities of Secondary Symptoms.*—By M. RICORD of Paris—In the report of M. Ricord's lectures, it is stated as the result of his extensive experience in the Venereal Hospital, that he has arrived at the following conclusions relative to the chances of secondary symptoms after a primary sore.

1. Primary ulcer is the indispensable precedent of secondary syphilis; without chancre there can be no general infection, except in the rare case of hereditary disease.

2. Simple non-indurated chancre and gangrenous chancre are very seldom, and only in exceptional cases, followed by secondary syphilis.

3. Indurated chancre always gives rise to constitutional infection.

4. The seat of chancre does not in the least degree influence the production of secondary symptoms. Provided the chancre be indurated, secondary disorder is as common and as constant after sores of the mouth, hand, nose, or foot, as after chancres of the penis.

5. The size of a chancre, the number of sores a person is affected with, do not increase his chances of general infection, always provided none be indurated.

6. If the primary sore be destroyed during the six first days of its existence, no secondary symptoms will follow.

7. If six months elapse after the cure of a chancre (no mercury having been exhibited,) without the appearance of secondary eruptions, all fear of constitutional symptoms may be laid aside.—*Ibid. from the Prov. Med. Jour.*

